# Healthy older adults balance pattern under dual task conditions: exploring the strategy and trend

**DOI:** 10.15171/hpp.2016.34

**Published:** 2016-10-01

**Authors:** Bahareh Zeynalzadeh Ghoochani, Seyed Ali Hosseini, Saeed Talebian, Akbar Biglarian, Afsaneh Zeinalzadeh, Salman Nazary-Moghadam, Seyed Alireza Derakhshanrad

**Affiliations:** ^1^Department of Occupational Therapy, School of Rehabilitation, University of Social Welfare and rehabilitation Sciences, Tehran, Iran; ^2^Social Determinants of Health Research Center, University of Social Welfare and Rehabilitation Sciences, Tehran, Iran; ^3^Department of Physical Therapy, School of Rehabilitation Sciences, Tehran University of Medical Sciences, Tehran, Iran; ^4^Department of Biostatistics, University of Social Welfare and Rehabilitation Sciences, Tehran, Iran; ^5^Department of Physical Therapy, Faculty of Rehabilitation Sciences, Tehran University of Medical Sciences, Tehran, Iran; ^6^Department of Physical Therapy, School of Paramedical Sciences, Mashhad University of Medical Sciences, Mashhad, Iran; ^7^Occupational Therapy Department, Shiraz University of Medical Sciences, Shiraz, Iran

**Keywords:** Balance, Elderly, Stroop test

## Abstract

**Background:** In line with health promotion plans, early intervention and fall prevention in geriatric population, it is important to study healthy individuals balance mechanisms. The aim of this research was to investigate the effect of adding and removing visual input and dual task on elderly balance.

**Methods: ** Twenty healthy elderly recruited from four different senior citizen health club centers and from the University of Social Welfare and Rehabilitation Sciences (USWR) participated in this analytic cross-sectional study. At USWR’s Motor Control Laboratory, the participants’ postural sway were assessed using force plate in 4 distinct double leg standing conditions with and without presence of visual input and Stroop dual task. Postural and Stroop variables were compared.

**Results:** Findings indicated that when the elderly encountered with either dual task or absence of visual input, they can still manage the situation in a way that changes in sway parameter would not become significant. But, when these two conditions occurred simultaneously, the participant’s balance strategy fluctuated. Therefore, the mean velocity showed a significant difference between the “single quiet standing” condition and the condition of standing with eyes closed while the participants were answering Stroop dual task (Mean difference = -0.007, 95% CI = -0.012, -0.002).

**Conclusion:** It appears that velocity parameter is sensitive to small changes, so it is recommended that researchers include this parameter in their future analyses. Balance in elderly can be manipulated by dual task and visual input deprivation.

## Introduction

Everyday life postural tasks are not separated from other tasks such as cognitive ones. These concurrent performances are called “dual task,” and require part of attentional capacity, leaving less capacity for other task. Simultaneous division of additional capacities may have dual task cost.^1^ As a general view, senior citizen population have some kind of age-related decline in postural task performance while they perform other task.^1^ It has been illustrated that balance performance during dual task can be a reliable source of information to differentiate between elderly with or without history of fall.^2^ Consequently, if dual task is a good method for prompt detection risk of fall, the best choice to fulfill such assessment is force plate. This device shows subtle and small changes in millimeter and centimeter which is best suited for fall prevention studies. Since every macro change starts at micro level, therefore, prevention plans should begin when it happens or even prior to the problem that takes shape invisibly or in an unrecognizable and undetectable manner. Fortunately, center of pressure helps to detect what is happening before its clinical manifestation.^3^ In interpreting COP patterns, it is widely believed that lower sway means better balance^4,5^; however, other investigators have shown that Parkinson disease is an exception to the rule.^6,7^ During the past decades, nonlinear variables have been emerged to assess complexity of balance and manage the existing conflicts amongst researchers.^8^ So it is particularly helpful to study linear alongside nonlinear variables which can lead to more trustworthy, credible and reliable interpretations.

On the other hand, another important issue in elderly balance is weighting sensory inputs. Some evidences suggest that some type of alteration occur in sensory assignments during aging process,^5,9,10^ and it has been hypothesized that decreased contribution of sensory inputs would lead to disturbed balance.^11^

Overall, it is vital to check healthy individual balance behavior to see how they would cope with upcoming challenges, exploring the substrate mechanisms that offer more data which can be applied in health promotion plans and perspective. Knowledge about how their balance would be affected by their vision contribution and external imposed unfamiliar dual task, would help to detect their achilles heel which can be reinforced, making them prepared for future real life context and challenges beforehand. Hence, this paper was aimed to study: balance during double leg quiet standing by adding and removing auditory Stroop (cognitive dual task) and visual inputs, combining two phenomena of sensory input and divided attention to explore the substrate mechanism, reaction patterns and strategy trend in individuals entering the senile period.

## Materials and Methods

### Participants

Having read the announcement posted in the municipality of Tehran (Zone 2) in four different senior citizen health club centers and University of Social Welfare and Rehabilitation Sciences (USWR), Tehran, Iran, some volunteers took part in this analytic cross-sectional study in August, 2015. According to sampling formula, 20 qualified participants meeting inclusion criteria were recruited. This group included 14 males and 6 females with the mean age of 61.15 (SD: 1.95) years, height of 166.9 (SD: 7.07) cm, weight of 73.9 (SD: 8.89) kg, and body mass index (BMI) average factor of 26.6 (SD: 2.8). Their Mini–Mental State Examination (MMSE) mean score was 28.25 (SD: 1.97), indicating normal cognitive function. Inclusion criteria were: living independently, active member of society and being capable of understanding and following the instructions that they were given.

The exclusion criteria were: history of fracture in spine or lower extremity, seizure or any kinds of epilepsy diagnosed by a neurologist, known motor impairments or movement-related disorders, mental or psychological problems, taking sedative drugs or any medicine affecting consciousness, having diabetes, any kind of speech impediment, visual and auditory impairment, and any vestibular or sensory problem. There was an important emphasis that the subjects were not considered as ‘frail’ individual. Prior to the study, participants were informed of test’s objectives and procedures and they were asked to fill out a written inform consent.

### Procedures 

For the sake of postural task, participants were asked to stand barefooted on force plate (Kistler [Switzerland] - sampling rate 400 Hz - sensitivity10 for the duration of 20 seconds) looking straight forward, arms resting on their sides with 4 levels of difficulty (eyes open or closed – with or without Stroop task). The orders of these four conditions were randomized, in a manner in which to avoid learning. To increase the force plate accuracy it was calibrated before each test. Study setting was located in motor control laboratory at the USWR, Tehran, Iran.

Participants were asked to stand at the center of a force plate with a small gap between their feet. Measurements were repeated in four different conditions where balancing system was challenged.

To perform “double leg eyes open” (condition A) individuals were asked to stand on a force plate with their eyes open without cognitive task for 20 seconds. They were asked to look forward in silent without any movement or any unusual deep inhale or exhale. They were asked not to shift their weight from one leg to another. Their COP sway was documented.

To execute “double leg eyes open with Stroop dual task” (condition B), participants stood on the force plate and when they became stable the auditory Stroop test (cognitive test) was initiated. Auditory Stroop test took 500 milliseconds to begin and continued for 20 seconds. This condition simulated a real life situation in which a person may have to perform a cognitive task while maintaining his/her balance.

For accomplishing “double leg eyes shut” (condition C), Individuals were asked to stand on a force plate with their eyes shut without cognitive task for 20 seconds. This condition assessed sway patterns when visual input was removed to deprive a person of visual inputs simulating real life condition. This test explored the effect of one major sensory source being absent.

“Double leg eyes shut Stroop dual task” (condition D) is the combination of eyes shut and the dual task design to increase the level of difficulty, to examine whether the subject balance system can cope with the imposed task.

These four conditions were performed in random order to avoid the learning effect. The procedure comprised of becoming familiar with cognitive auditory Stroop dual task and four assessment conditions. Initially, three pilot trials were performed playing Stroop sounds in sitting position since participants were unfamiliar with this task. In order to perform Stroop test, words ‘high’ and ‘low’ were played in either high or low tone pitch.^12^ Participants were asked to repeat the tone pitch as quickly as possible ignoring the played word itself. For instance, if the word high was produced in low tone pitch, the correct answer was “low” and vice versa. The congruency between the word and tone was randomized. Error ratio (numbers of error divided by total), and average reaction time (delay in answering after stimulus) were recorded for further calculation.^13^ Onset of stimuli was after 500 ms, two consecutive stimuli interval were randomly switched from 2000 ms to 3000 ms in order to avoid anticipatory answers.^14^ Auditory Stroop task was implemented via wireless earphone and microphone (LEM-NP10, Taiwan) and soft-wares utilized were R2015a Matlab program, Microsoft Office Excel 2013 and IBM SPSS statistics 20.

### Measures

Present study had two groups of dependent variables; one was related to postural data acquired by force plate and another group of variables were those related to cognitive dual task performance. Postural variables were center of pressure displacement parameters (quantitative variables calculated by Matlab software), namely; mean velocity (MV), area, displacement in X axis i.e. range side way (RSW), and range of anterior posterior displacement in Y axis (Range Fore After; RFA) linear variables and two non-linear variable of entropy X and entropy Y. Second group of variables regarding cognitive dual task performance were reaction time and error ratio. The independent variables were visual input and Stroop task during double leg quiet standing.

### Statistics

For data analysis, two statistical tests were performed separately; the repeated measurement analyses of variance for postural parameters and an extra paired *t* test on dual task performance variables. Statistically significant differences were calculated at significant levels of 0.05 and 0.1. Kolmogorov-Smirnov test *P* values were higher than 0.05 for COP and Stroop variables that proved the normal distribution. Homogeneity assumption was tested by Mauchly test of sphericity.

## Results

Repeated measurement identified a significant difference in velocity parameter (Wilks’ lambda=0.63, F [3, 17] = 3.325, *P* = 0.045). In succession to pervious measurements, Bonferroni pairwise comparison identified that mean of velocity between state A, D was significant at alpha=0.1 (*P* = 0.07), and according to LSD pairwise comparisons these two conditions were significantly different (*P* < 0.05) with 95% CI (-0.012, -0.002) (Table 1).

According to paired *t* test comparing cognitive performance, there was no statistical significant difference between eyes open and shut (reaction time for eyes open condition 1.22 [SD: 0.37] and eyes shut 1.06 [SD: 0.26]; t [19] = 1.589, *P* = 0.129 - error ratio during eyes open trial 0.21 [SD: 0.054] and eyes shut 0.20 [SD: 0.059]; t [19] = 0.551, *P* = 0.588).

**Table 1:** Results of repeated measurement analysis of variance for postural variables

**Variables**	**A- Double leg eyes open**	**B- Double leg eyes shut**	**C- Double leg eyes open with dual task**	**D- Double leg eyes shut with dual task**	**Differences between conditions**
Mean velocity^a^	2 ± 0.007	2.5 ± 0.006	2.5 ± 0.012	2.7 ± 0.008	Between condition A and condition D
Area	2.7 ± 1.91	3.8 ± 2.92	4.3 ± 3.70	2.84 ± 2.58	
RFA	2.50 ± 0.95	2.83 ± 0.78	2.99 ± 1.31	2.79 ± 1.40	
RSW	2.451 ± 1.59	2.20 ± 1.15	2.83 ± 2.34	2.13 ± 1.53	
Entropy X	0.22 ± 0.23	0.26 ± 0.24	0.21 ± 0.20	0.13 ± 0.19	
Entropy Y	0.181 ± 0.19	0.189 ± 0.21	0.101 ± 0.11	0.131 ± 0.18	

Unit: COP Area = CM^2^, RFA and RSW = CM, MV = CM/S. ^a^ *P *< 0.05.

## Discussion

Statistical results according to repeated measurement analysis of variance test showed that there was a significant difference between means of single task eyes open (condition A) and dual task with eyes shut (condition D), in velocity parameter. Based on the current study amongst the four mentioned conditions, balance pattern changed considerably when visual input was eliminated accompanied by imposed cognitive auditory dual task in comparison with the time when eyes were open performing only postural task of double leg standing. Specifically, our results propose that elderly strategy to maintain their center of mass within their base of support and mastery in controlling sway is to alter the MV at the onset of aging process. But there were no significant differences in other sway variables.

MV is a dynamic parameter, an index for postural control in parallel to area, RFA and RSW that are indices of postural stability. The most important variable in this article to be discussed is the mean velocity due to its’ statistical significant difference. Kuznetsov and Riley suggested that COP parameters based on velocity are adequate indices in assessing balance performance while silent standing.^15^ Raymakers et al presented mean displacement velocity as the most revealing variable in most circumstances.^16^ Our findings are in accordance with Raymakers et al, since the only variable sensitive to changes by manipulating visual input and dual task was velocity giving information about chosen tactics.

With respect to quantity, MV hit the highest point during eyes shut condition answering Stroop test (condition D) when compared with other conditions. However, it fell to the lowest point during the eyes open single task condition (A). Thus, a sharp difference existed between these two trials. The possible explanation for this sharp and noticeable change could be the collective impact of sensory deprivation and dual task cost. Regarding the other two conditions (B and C) in which only one independent variable was manipulated, MV had increased slightly in comparison with eyes open single task (condition A) in an insignificant manner, and their fairly equal mean amounts plateaued and leveled out at nearly 2.5 cm/s.

With regards to standard deviation of MV in four conditions it is noteworthy that there was a high value in dual task condition with open eyes (condition B), while the next highest amount was related to dual task condition with eyes shut (condition D) and eyes open single task (condition A). There was a sharp decline for MV variation of standard deviation in single task with eyes shut (condition C). The least standard deviation (variation) of single task condition with eyes shut can be ascribed to the concentration of individuals when their eyes were shut and the least distraction existed.

In terms of MV upward trend interpretation, it was in parallel with increasing difficulty. Traditionally it was assumed that increase in sway parameters was related to poor balance,^17^ assuming that this hypothesis is valid, elderly balance is weaker when confronted with dual task eyes shut and postural task is sacrificed to accomplish cognitive dual task giving the priority to Stroop. Considering the existing exceptions of freezing phenomenon and decreasing sway variables in individuals with Parkinson,^7,8^ and according to controversies claimed by van Emmerik and van Wegen^8^ who counted decreased parameters related to pathology or aging, there is an uncertainty about judging or labeling good or bad balance based on increased or decreased sway variables. Therefore, the new chosen strategy is not necessarily good or bad, in fact, it is good or even the best choice for the individual confronting balance challenge whose balance may be lost if the strategy were taken from him. Perhaps, it is the best strategy adopted by an individual current condition that is chosen by the intelligence of human motor control. It is not farfetched to say that this trend only shows the best tactic chosen by individual to overcome his/her limitations, as their best choice. When the time comes to combine visual input and dual task, the individual shows change in MV as a substrate latent mechanism to maintain the equilibrium. As a result change in strategies occurred, to control subsequent displacement, that is why no significant changes in displacement parameters were observed. On the contrary to other studies that claimed high velocity as an indicator of poor balance, in this study, increased velocity was presented as the underling compensatory solution to control displacement which is the indicator of conscious well-run balance system that only change when the system was overloaded by multiple changes. This tactic enabled our elderly subjects to maintain their center of gravity enabling them to react faster and return to the sway starting point, with simultaneous accomplishment of tasks interference and setting their priorities.

Horak discussed the association of visual, vestibular and somatosensory inputs during quiet standing and their compensatory weight adjustment when external or internal elements were altered.^18^ In this research, visual contribution had to be compensated with somatosensory, vestibular system and other elements affecting balance, so that sway was not significantly different for closed eyes single task trial (condition C). Dual task performance has also been reported to effect sway in a significant manner in elderly with the history of falling,^2^ but in this research when individuals were encountered with just concurrent dual task (condition B) no significant differences in sway was observed. This article findings postulate that participants were stable enough to manage the sensory deprivation challenges one at a time and dual task, separately. It seems that their balance had not yet become bendable when encountering minimal changes. Hence, our results are similar to the concept that mammalian brain at the beginning of aging period may experience some kind of advanced changes such as dendritic proliferation.^19^

The latest notion is to describe the existing pattern as well as the nonlinear variables.^8^ These parameters emerged to assess complexity nature of balance due to their trustworthiness, credibility and reliability. Entropy decreased by hardening the trials, but it was not statistically significant so it is encouraging that although MV had increased by task difficulty, but by adding nonlinear variable interpretation, individuals have showed somewhat stable balance behavior when comparing all four conditions from statistical perspective.

So we can conclude that, if entropy did not change significantly, it can be assumed that their balance complexity was not manipulated by circumstances which supports the increased velocity as a tactic for maintaining balance. This is in favor of presenting dual task eyes shut condition with increased MV as normal. Other issues that have to be considered are aging stage; early, middle and advanced.^19^ Our study mainly focused on the early stage and the results could only be denoted for healthy individuals.

With respect to dual task performance, there was no significant difference between eyes open and shut conditions (condition B and D). An important issue is the type of cognitive or postural activity being accompanied along with given directions.^2^ According to Pellecchia the effect on sway can be increased in parallel to dual task increased difficulty level.^20^ Swan et al reported dual task difficulty as the major contributor to sway fluctuation,^21^ and according to Polskaia et al if dual task helps to distract the attention from postural task it may improve balance, making it more automatic.^22^ Dual task used in the present article can be considered as unfamiliar and uneasy ones. In this research, individuals reacted faster in answering to Stroop stimuluses and their error ratios were lower, but not statistically significant when their eyes were shut (condition D) in comparison with open eyes condition (condition B). That may be attributed to possible concentration, being able to focus and organize their attention.

If early detection occurs in subtle balance changes in micro dimensions, it can help to prevent future macro changes. Some implications in practice and future prospect can be considered here. According to our findings, when a person goes through elderly phase his/her balance is not still necessarily and completely compromised by visual inputs or concurrent task, separately (condition B and C). But, in the condition D some small changes were found in velocity parameter. Similarly, Moghadam et al,^23^ found the mean velocity as one of the most reliable force plate parameters.

So after deep contemplation, it strike in mind that micro changes of balance would be early detected during more complicated and difficult conditions. The question is that if these primary changes start gradually and are not still dominant in the current study, can they be conquered even though are not tangible in real life context by practicing the combination of postural task with eyes shut and dual task which had been determined to be the probable achilles’ point. Last issue to consider about dual task paradigm is Bonnet’s idea on presenting synergy instead of duality^24^ when performing dual task, which in contrast to previous assumption of competing sources between two tasks. During this study dual task with closed eyes were used as assessment tools, but its application as an intervention can be investigated more in future studies. Dual task can be more effective when accompanied by special instruction^25^ although there is some evidence in which all COP parameters would not change by clues,^26^ in this research participants knew what would happen and when which implies the concept of anticipatory postural control as mentioned in the study of Shiravi et al.^27^

## Conclusion

According to our findings we offer some recommendations; Firstly, Velocity parameter is very sensitive to small changes in COP so it is better for researchers working with force plate to include this parameter in their future analyses. Entropy is also recommended to be accompanied by linear force plate variables to represent the complexity and hidden nature of balance conditions.

Secondly, in the light of current study, elderly balance performance were not very alterable as generally perceived to be during double leg stance. In the early stage of healthy aging, their balance on two feet would not be affected so much from visual deprivation or dual task separately, but when it came to combination of these two (i.e. dual task with eyes shut condition), a new strategy emerged to save their balance. The ensuing tactic of increased velocity is suggested to be assumed as a possible solution for an individual in controlling balance and self-organization during a tough condition; hence this is not an indicator of poor balance.

For the health care and promotion plans to be science-driven and evidence-based, balance exercises are suggested to be accompanied by “dual task with eyes shut” condition in clinics or even public sport since this condition was identified as an Achilles heel in this study making individual susceptible to changes in balance. Although this last suggestion should be investigated in future studies because dual task performance in this study was applied in order for balance assessment, so its indication as an intervention method and its durability was beyond the scope of this article.

## Limitation

The main limitation in this study was restrictions on using force plate; it is a very expensive device and is not available in any institute, so the research team was only permitted to work over a limited period of time in the USWR’s laboratory.

Finding can be generalized for healthy individuals entering elderly who are in good mental and physical condition. However, if these conditions are used for different groups with disabilities or ageing, most likely it will yield different results.

Conditions were designed according to the research question and thus the paper does not claim to cover all aspects of balancing; therefore, future studies should focus on other aspects of balance function such as dynamic tests in order to provide more information about the most challenging and risky situations.

## Future research

Since this study was not conducted based on random selections, future research with random sampling is recommended to be rid of selection bias. Frail, impaired or older elderlies can also be the target population of future studies but necessarily with considering in mind the safety precautions during balance assessment or intervention procedure preventing probable fall and subsequent damages during balance research.

## Acknowledgments

Special thanks goes to Pezhman Lali for his major contribution in writing Persian version of auditory Stroop test program, University motion laboratory manager Mrs. Nabavi and Ailin Talimkhani for her great contributions during data gathering process and authors would like to thank Mr. Argasi for his efforts into English language editing. Although no financial support was received especially for the thesis, we would like to thank the USWR of Tehran, Iran. 

## Ethical approval

This project was approved in University of Social Welfare and Rehabilitation Sciences in partial fulfillment of PhD thesis and ethical committee has accepted the test conditions as being proper to be performed on human subjects.

## Competing interests

Authors declared no potential conflicts of interest.

## Authors’ contributions

BZG, SAH, ST, AZ, and SNM have made contributions to the study conception and design. BZG has made contributions to acquisition of data. All authors have made contributions to analysis and interpretation of data. In addition, they have contributed to the drafting and revision of the article, and the approval of the final version.
